# Phase separations in oncogenesis, tumor progressions and metastasis: a glance from hallmarks of cancer

**DOI:** 10.1186/s13045-023-01522-5

**Published:** 2023-12-18

**Authors:** Le-Wei Zheng, Cui-Cui Liu, Ke-Da Yu

**Affiliations:** Department of Breast Surgery, Department of Oncology, Key Laboratory of Breast Cancer in Shanghai, Cancer Institute, Fudan University Shanghai Cancer Center, Shanghai Medical College, Fudan University, Shanghai, 200032 China

**Keywords:** Biomolecular condensate, Membrane-less organelle, Cancer, Novel therapeutics, Liquid–liquid phase separation

## Abstract

Liquid–liquid phase separation (LLPS) is a novel principle for interpreting precise spatiotemporal coordination in living cells through biomolecular condensate (BMC) formation via dynamic aggregation. LLPS changes individual molecules into membrane-free, droplet-like BMCs with specific functions, which coordinate various cellular activities. The formation and regulation of LLPS are closely associated with oncogenesis, tumor progressions and metastasis, the specific roles and mechanisms of LLPS in tumors still need to be further investigated at present. In this review, we comprehensively summarize the conditions of LLPS and identify mechanisms involved in abnormal LLPS in cancer processes, including tumor growth, metastasis, and angiogenesis from the perspective of cancer hallmarks. We have also reviewed the clinical applications of LLPS in oncologic areas. This systematic summary of dysregulated LLPS from the different dimensions of cancer hallmarks will build a bridge for determining its specific functions to further guide basic research, finding strategies to intervene in LLPS, and developing relevant therapeutic approaches.

## Background

The spatial and temporal coordination of biochemical reactions is crucial for cellular physiology [1]. While membrane-bound organelles are essential for spatially organized cellular processes, the discovery of membrane-less organelles (MLOs) has shed light on new mechanisms for tightly controlling processes within cells [2]. MLOs, as known as biomolecular condensates (BMCs), include the nucleolus [2], Cajal bodies [3], nucleoli [4], stress granules (SGs) [5–7], and super-enhancers (SEs)[8–10] etc. These structures typically range from 0.1 to 3 µm [11]and play key roles in facilitating or modulating specific cellular processes. BMCs and MLOs are both formed by the process of phase separation, and in most scenarios, these two concepts are equivalent.

Until the emergence of the concept of liquid–liquid phase separation (LLPS), the formation and organization of MLOs remained unclear [12]. Thus, LLPS provides a reasonable framework to explain the formation mechanism of MLOs and BMCs. This dynamic process involves the transition of biomolecules from a homogeneous environment to sparse and dense phases [11, 13, 14], aiming to reach the lowest-entropy state. Notably, LLPS occurs when multivalent biopolymers instantaneously interact with each other [15–17], forming liquid-like entities such as bodies, puncta, granules, droplets, and condensates [18].

Normal BMCs ensure basic cellular functions, whereas their aberrant forms result in cellular dysfunction and possible tumorigenesis. Studies have demonstrated that LLPS are crucial in the regulation of tumor onset, progression [19], including promoting cancer cells proliferations and metastasis. Further, the hallmarks and enabling characteristics of cancer in the 2022 version provide a framework for further oncological studies[20]. However, understanding of the regarding phase separation processes involved in each hallmark is still limited. Therefore, unveiling a novel dimension of its biological functions is in need.

In this review, we include all cutting-edge and typical articles related to liquid–liquid separation in oncology. Firstly, we describe the methods used to investigate LLPS, followed by their role in promoting the formation of BMCs/MLOs. Subsequently, we examine the current understanding of how LLPS influences tumorigenesis, progression and their emerging role in cancer treatment. Finally, we comprehensively summarize the latest insights into methods to interfere with aberrant forms of BMCs.

## Mechanisms and methods associated with the phenomenon of LLPS

### Concepts and mechanisms

**Fig. 1:**
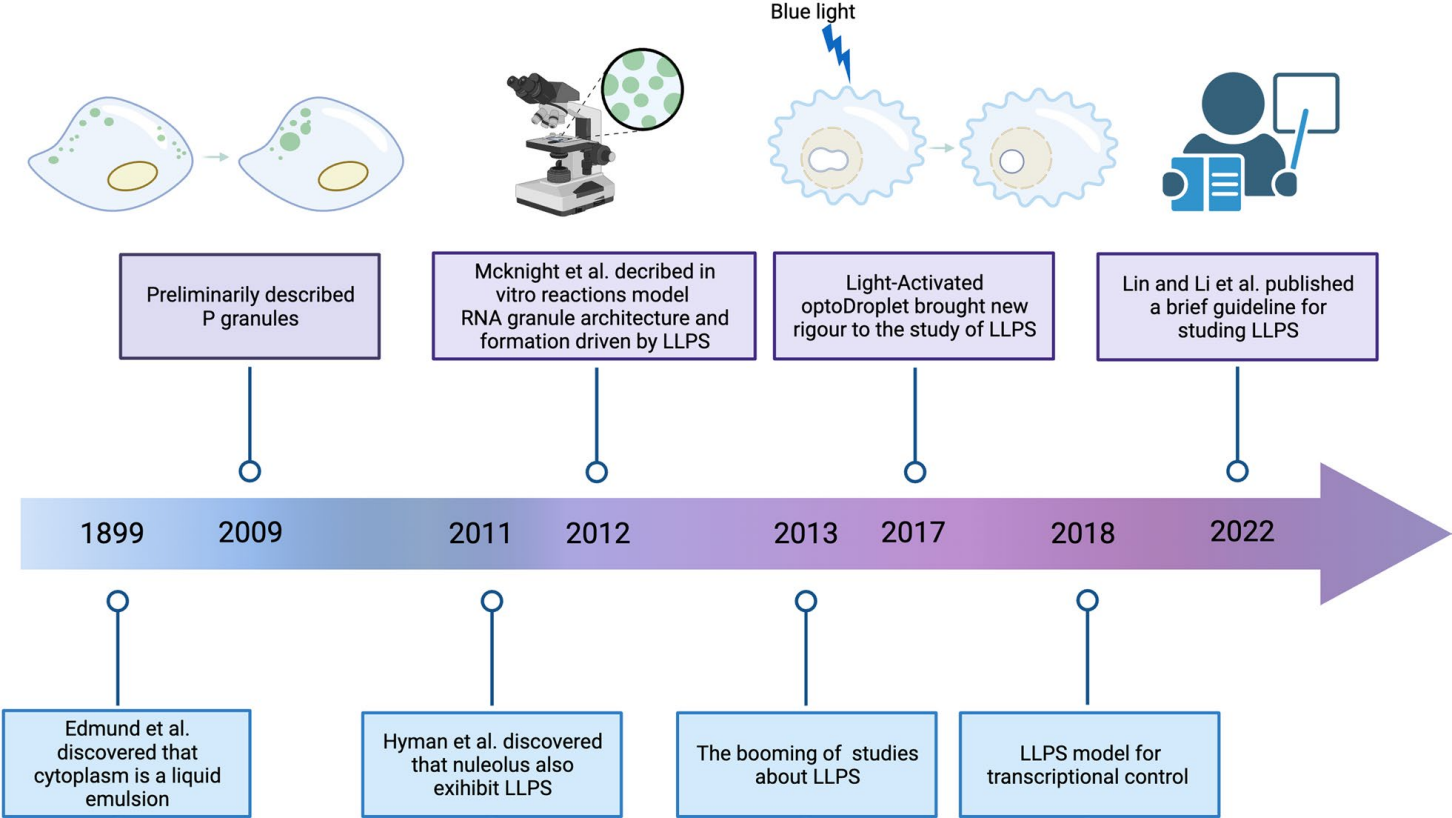
History of LLPS research developments. Milestone discoveries are outlined

**Fig. 2:**
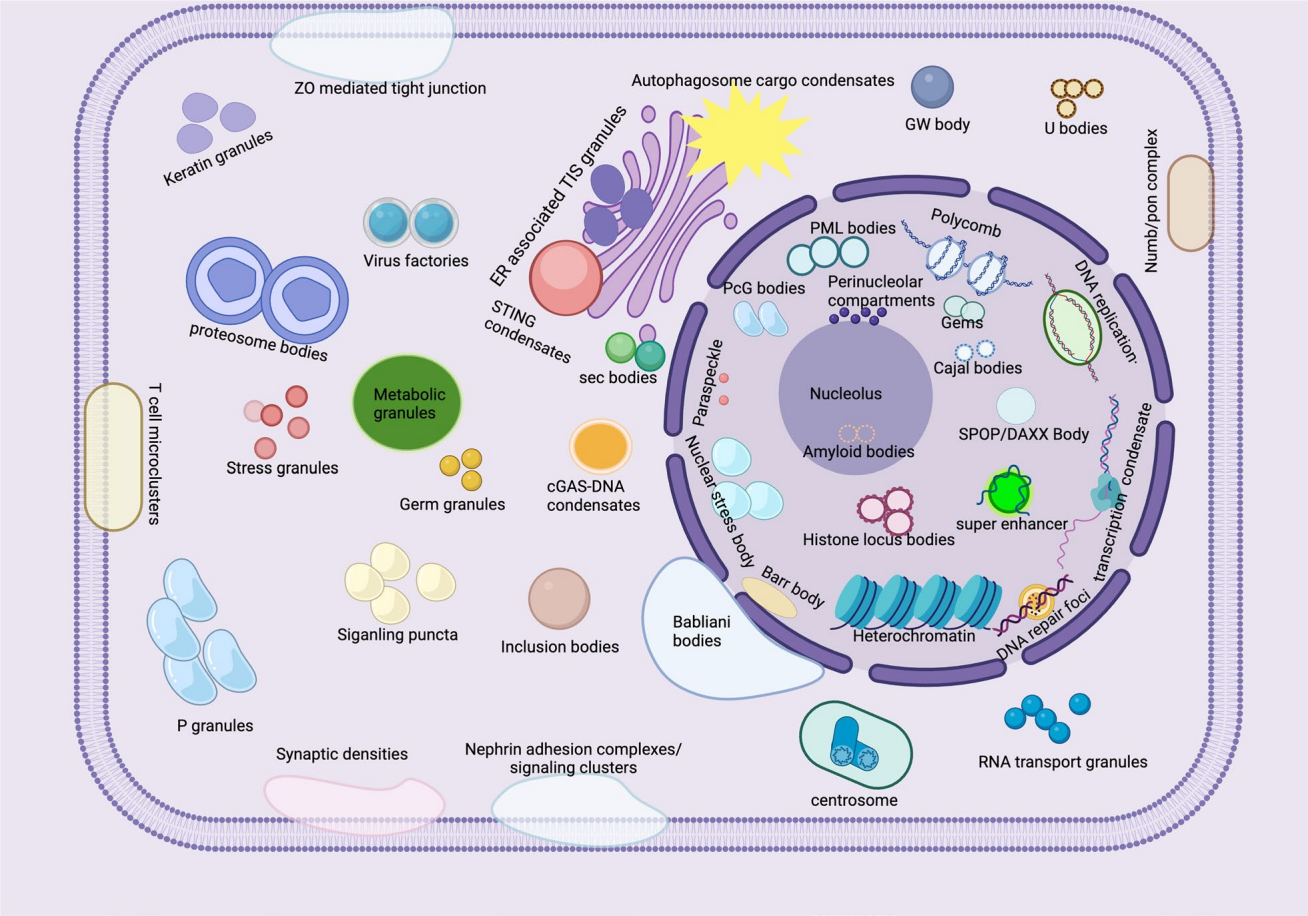
Intra-cellular MLOs within a eukaryotic cell. MLOs are distributed in the nucleus, nuclear membrane, cytoplasm, and plasma membranes of cells. Nucleolus, perinucleolar compartments, paraspeckles, Cajal bodies, transcription condensates, Gems, DNA repair foci, nuclear stress bodies, PcG bodies, histone locusbody, PML bodies, DNA replication bodies, polycombs, SPOP/DAXX bodies, super enhancers, heterochromatin, and amyloid bodies (located in nucleolus) are located in the nuclear by the LLPS. Whereas some MLOs are distributed in the nuclear membrane (Babliani bodies), cytoplasm (such as sec bodies, cGAS-DNA condensates, ER associated TIS granules, autophagosome cargo condensates, stress granule, P granules, U bodies, Virus factories, Numb/pon complex, RNA transport granules, centrosome, inclusion bodies, siganling puncta, GW bodies, germ granules, transport RNP, and proteosome bodies, metabolic granules, keratin granules), and cell plasma membrane (such as immune synapse densities, Numb/pon complex, Nephrin adhesion complexes/ signaling clusters, T cell microclusters, and ZO mediated tight junction)

Phase separation is defined as the spontaneous aggregation of molecules when their concentration exceeds a certain threshold, thus forming a membrane-less compartment [21]. Typically, the interactions between macromolecules in LLPS are typically non-covalent and of low affinity [22, 23]. This process is often driven by the modification of intrinsically disordered regions (IDRs) within proteins [24, 25]. The concept of LLPS was first introduced in the biochemical field of biochemistry in 2009 by Hyman and colleagues with various milestone events followed subsequently (Fig. 1), offering a novel perspective on various MLOs distributed in cells (Fig. 2) [26]. Although several in silico tools help forecast the potential of phase-separated molecules (Table 1), comprehensive summaries of the characteristics and conditions that induce LLPS are limited.

**Table 1:** Overview of databases related to liquid–liquid phase separation (LLPS)

Category	Database	Availability	Details of databases	References
Prediction of LLPS related proteins	SGnn	http://sgnn.ppmclab.com	Proteins bearing prion-like domains (PrLDs)	[27]
	PhaSepDB	http://db.phasep.pro/	Phase-separation related proteins	[28]
	D^2^P^2^	https://d2p2.pro/search	Phase-separation related proteins	[29]
	PLAAC	http://plaac.wi.mit.edu/	Prion-Like Amino Acid Composition	[30]
	DrLLPS	http://llps.biocuckoo.cn/	Proteins in this database are classified as drivers, regulators and potential Clients	[31]
	PhaSePro	https://phasepro.elte.hu	A manually curated database of LLPS driver proteins in various organisms, with emphasis on the biophysical properties that govern phase separation.	[32]
	BioGRID	https://thebiogrid.org/	Database of Protein, Genetic and Chemical Interactions	[33]
	LLPSDB	http://biocomp.ucas.ac.cn/llpsdb/home.aspx	A database of proteins undergoing LLPS in vitro	[34]
	HUMAN CELL MAP	https://cell-map.org/ or https://humancellmap.org/	Summarizes for each compartment the enrichment of expected domains and motifs as well as GO-terms Provides channels to analyze spatiotemporal correlations between proteins in different organelles	[35]
	MLOsMetaDB	http://mlos.leloir.org.ar/	Unified resource of MLOs and LLPS associated proteins	[36]
	catGRANULE	http://s.tartaglialab.com/	A website good at predicting LLPS propensity of dosage-sensitive proteins	[37]
	PScore	https://github.com/haocai1992/PScore-online#pscore-online	A machine learning algorithm that predicts the likelihood of phase separated proteins	[38]
Prediction of LLPS related RNAs	RPS	http://rps.renlab.org/#/Home	A comprehensive database of RNAs involved in liquid–liquid phase separation	[39]
	RNAPhaSep	http://www.rnaphasep.cn/#/Home	A resource of RNAs undergoing phase separation	[40]
	RNA granule database	http://rnagranuledb.lunenfeld.ca/	A database containing RNA granules	[41]
Integreation of LLPS related diseases	DisPhaseDB	http://disphasedb.leloir.org.ar	An integrative database of diseases related variations in liquid–liquid phase separation proteins	[42]
Prediction of specific structures or features of LLPS	IUPred2A	https://iupred2a.elte.hu/	Combination of the iupred database and the ANCHOR database, which can predict the disordered and disordered binding regions of proteins	[43]
	PONDR	http://www.pondr.com	Predictor of natural disordered regions	[44]
	MobiDB	https://mobidb.org	Provides information about intrinsically disordered regions and related features	[45]
	CIDER	http://pappulab.wustl.edu/CIDER/	Calculation of many different parameters associated with disordered protein sequences	[46]
	ZipperDB	https://services.mbi.ucla.edu/zipperdb/	Predictions of fibril-forming segments within protein	[47]
	Metadisorder	http://iimcb.genesilico.pl/metadisorder/	Prediction of protein disorder	[48]
	DisMeta	https://montelionelab.chem.rpi.edu/dismeta/	Prediction of protein disorder	[49]
	Expasy	https://web.expasy.org/compute_pi/	Computation of the theoretical pI (isoelectric point) and Mw (molecular weight)	[50]
	AMYCO	http://bioinf.uab.es/amycov04/	Evaluation of mutation impact on prion-like proteins aggregation propensity	[51]
	MFDp2	http://biomine.ece.ualberta.ca/MFDp2/	Accurate sequence-based prediction of protein disorder which also outputs well-described sequence-derived information that allows profiling the predicted disorder	[52]

### Structural characteristics and critical components that triggers LLPS

The concept of a driver (or scaffold)/client is widely accepted. Proteins, DNA, and RNA can also be used as scaffolds. With multiple binding sites, these macromolecules facilitate weak interactions and trigger LLPS. The detailed structures are summarized below.

#### Multi-foldable domains

**Fig. 3:**
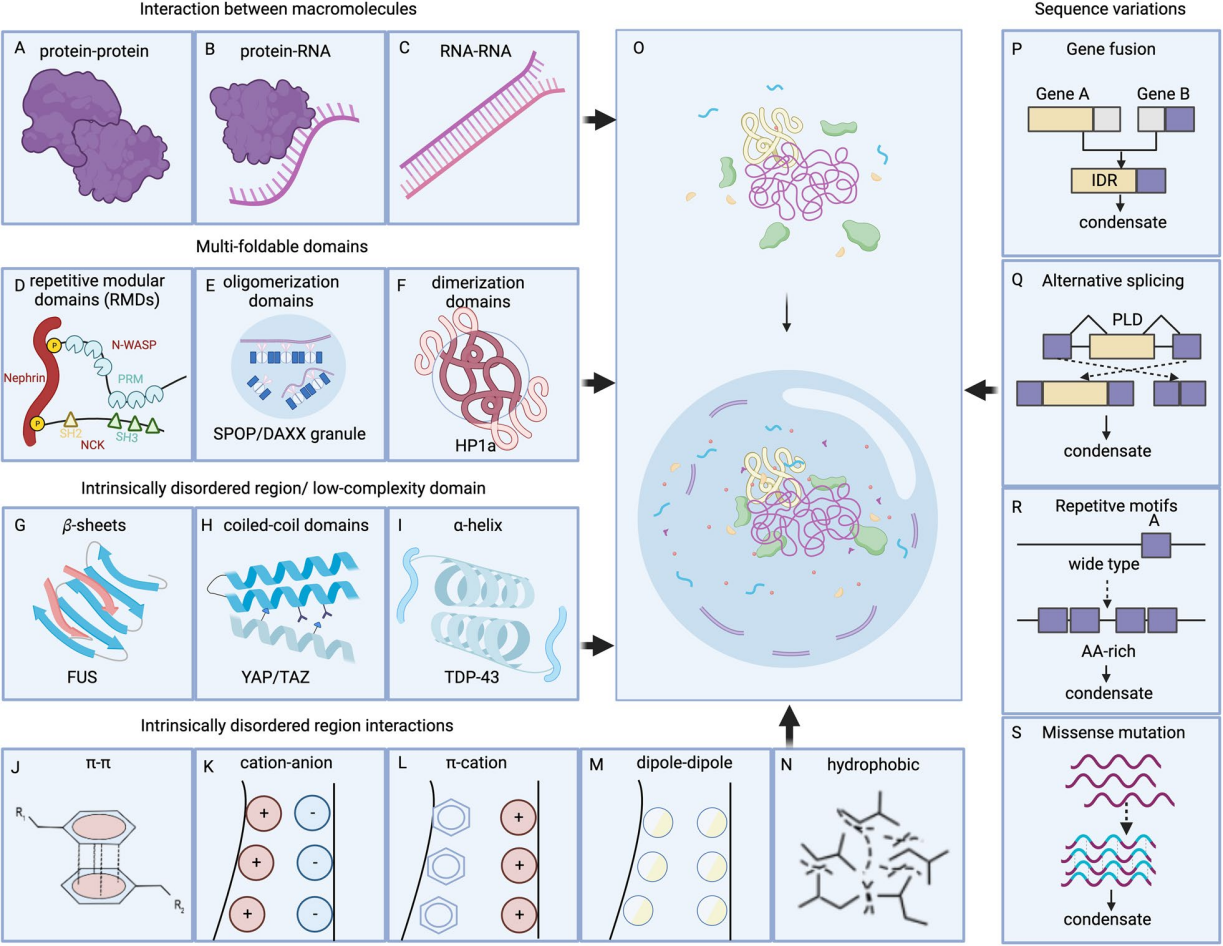
Basic condensates promoting features. **A**-**C** Interactions between macromolecules that facilitate phase separation. **D** The SH2 domain of NCK binds to Nephrin, and NCK possesses three SH3 domains that can bind the proline-rich motifs (PRMs) of N-WASP, showing a typical repetitive molecular domain (RMD) that contributes to LLPS. **E** Oligomerization of SPOP and its interactions with substrates can induce phase separation. **F** Dimerization of HP1a promotes LLPS. **G**–**I** Several classic IDRs, which consist of LCDs. **J**–**N** Fundamental interacting force between IDRs. **O** Formation of BMCs, from dissociation to assembly. **P**–**S** Four types of sequence variations that drive phase separation

One of the most common structural features that facilitates LLPS is multivalency, which involves the interaction of various macromolecules (Figs. 3A–C). By using multiple, similar domains to mediate the interactions, these macromolecules effectively trigger LLPS and attract client molecules to form condensates. For example, the proline-rich motif (PRM) domain characteristic of the neural Wiskott-Aldrich syndrome (N-WASP) interacts with the SH3 domain of NCK, thereby inducing LLPS [53]. A similar principle applies to the nephrin/Nck/N-WASP system, wherein the phosphotyrosines of nephrin interact with the SH2 and SH3 domains of NCK to bind to the PRMs (Fig. 3D) [54]. Similarly, higher-order polymerized structures are formed via the tandem dimerization domains of the speckled POZ protein (SPOP) and its interaction with cullin-3-RING ubiquitin ligase and other substrates, promoting its localization in nuclear speckles[55] (Fig. 3E). Dimerization or oligomerization of proteins can also contribute to LLPS. For example, when the dimerization of HP1a is disrupted, the mobility of the droplets increases, hindering the maturation of heterochromatin formations (Fig. 3F)[56].

#### IDR/low-complexity domains contribute to LLPS

IDRs are distinctive features of certain proteins of the condensates, accounting for 33–55% of eukaryotic proteomes [57, 58]. Like IDRs, low-complexity domains (LCDs) are also distinctive features of proteins comprised by highly biased amino acid compositions [59]. IDRs and LCDs lack stable tertiary structures and have flexible conformations, making them prone to undergo LLPS [11, 60–62]. β sheets in TDP34/FUS (Fig. 3G), coiled-coil domains in YAP/TAZ (Fig. 3H) and alpha-helix in TDP43 (Fig. 3I), exemplify the role of LCDs in LLPS [63–69]. While IDR interactions involve pi-pi interactions, salt bridges between opposing charge residues, pi-cation interactions, dipole-dipole interactions (Van der Waals forces), and hydrophobic forces (Figs. 3J–N) represent different forms of LLPS [70].

#### Nucleic acids regulate LLPS

Nucleic acids, especially the single-stranded nucleic acids, tend to aggregate to form droplets, whereas double-stranded nucleic acids tend to form gel-like aggregates [71]. Via electrostatic interactions and the pairing of repeating molecules, certain RNAs achieve polyvalency, effectively inducing LLPS in combination with proteins, as observed in the RG/RGG-rich domains of the SERPINE1 mRNA-binding protein 1 (SERBP1) system [72]. In contrast, the RNA concentration does not show a strong positive correlation with the phase transition ability [73]. RNA modifications and non-coding RNAs can also induce LLPS spontaneously [74] or by attaching to proteins, facilitating clients recruitment for the condensate assembly [75–77].

#### Head-to-tail polymerization

Occasionally, stable structural domains in proteins, such as SAM and DIX, retain their ability to trigger local condensation [78, 79]. Among the dishevelled and axin components of the Wnt signaling, the DIX domain can assemble in a head-to-tail manner and promote Wnt signaling [80, 81]. The SAM domain of the tankyrase protein forms similar puncta in a head-to-tail manner to bind and ribosylate poly ADP AXIN, thus promoting Wnt signaling [82]. These structural conditions facilitate the formation of condensates (Fig. 3O).

#### Sequence variations at the gene levels

Disease-related genomic changes regulate LLPS. The NUP98 fusion protein in leukemia, carrying IDRs, serves as a good model for gene fusion [83] (Fig. 3P). Similar results have been obtained with anaplastic lymphoma kinase (ALK) and BCR-ABL1 fusions [84, 85]. Linear motifs that modulate ligand recognition within IDRs control the function of alternatively spliced (AS) proteins [86, 87] and modulate their assemblies (Fig. 3Q). On the contrary, repetitive motifs can induce pathogenic repeat expansions (Fig. 3R). Missense mutations in IDRs and polymerization/modular domains may influence the phase transition status bilaterally (Fig. 3S). For instance, F291S and Y283S mutations in the heterogeneous nuclear ribonucleoprotein A2 scarcely affect the aggregation, whereas D290V and P298L mutations improve the condensation [88].

### External conditions and physicochemical properties affect LLPS

In this section, we focus on the conditions and the post-translational modifications (PTMs) which play a crucial role in regulating the dynamic transitions of molecules within the cell.

The interplay of various intracellular conditions, such as the concentration of proteins, pH level, and changes of the cellular milieu, alter the strength of polyvalent interactions. These conditions are key regulators of transitions within the cell. Furthermore, the concentrations of macromolecules are critical. When the concentration exceeds a critical threshold, the interaction between these macromolecules outweighs the forces that maintain homogeneity of the system, making the solution susceptible to phase separation. Conversely, when the concentrations are below this threshold, the components remain evenly distributed [89, 90]. The alterations of pH value can significantly impact LLPS by changing the surface charges of amino acids, the α-carbonyl groups, and the α-amino terminal protonation status. pH alterations affect the stability of specific proteins and change the secondary structure from ordered to disordered. Altering the protonation of amino acids directly influences the chemical properties of macromolecules, further altering their intermolecular interactions and triggering LLPS. For example, the decreased cytoplasmic pH, induced by external stimuli, can promote LLPS of naturally disordered proteins, as observed with Sup35 in yeast cells [91]. The increase in salt concentration and the addition of substances such as PEG3000 and glycerol can also modulate LLPS [73, 92]. Additionally, weak electrostatic interactions, driven by IDRs, are highly sensitive to changes in pH and ionic strength, potentially explaining LLPS induction due to environmental changes [17, 93]. In addition, temperature and stress levels can also trigger or disrupt LLPS by affecting the solubility of macromolecules [11]. Moreover, prion-like domains in proteins can sense pressure, influencing the solubility and phase behavior [94, 95].

The PTMs are crucial in the regulation of phase transitions by altering molecular interactions or directly modifying the potency of BMCs [96–98]. PTMs can induce changes of biomolecules in the spatial structures and state of proteins [96, 99]. PTMs of RNA-binding proteins (RBPs) can directly weaken or enhance the interactions between components, contributing to the formation of RNP granules, serving as an example of an MLO that is composed of RBP and RNA [96]. PTMs can promote or inhibit polyvalent interactions by influencing the condition of proteins, thus affecting the occurrence of LLPS [100]. Notably, the Lys residues within the IDRs are particularly prone to get SUMOylation, a modification that significantly contributes to the formation of the promyelocytic leukemia nuclear bodies (NBs). De-SUMOylation can lead to the release of a constituent protein and the separation of NBs during mitosis [101, 102].

Given the complexity of physicochemical conditions, the manipulation of PTMs is an intriguing approach to influence LLPS. Thus, it is pivotal to understand the possible mechanisms in cancer-related PTMs associated with LLPS (Table 2).

**Table 2:** Summary of cancer-related PTMs involved in LLPS

PTM	Disease association	Participants	Biological role	Regulation of LLPS	References
Ubiquitination	Non-small-cell lung cancer	USP42	Drives nuclear speckle mrna splicing and promote tumorigenesis	Promotion	[8]
	Multiple cancer types	p62	Promotes tumor cell survival by upregulating p62 liquid droplet formation and degradation	Promotion	[103]
	Multiple cancer types	SPOP/DAXX	Co-localizes with DAXX in Liquid Nuclear Organelles and facilitates DAXX Ubiquitination	Promotion	[104]
Phosphorylation	Multiple cancer types	TAZ	Formation of transcription compartment to promote gene expression	Promotion	[68]
Methylation	Leukaemia	YTHDC1-m6A condensates	Facilitates a phase-separated nuclear body and suppresses myeloid leukemica differentiation	Promotion	[105]
	Multiple cancer types	UTX (namely KDM6A)	Involved in chromatin-regulatory activity in tumour suppression	Promotion	[106]
Sumoylation	Colon cancer	RNF168	Genomic instability and DNA damage repair	Promotion	[107]
Acetylation	Multiple cancer types	KAT8-IRF1	KAT8-IRF1 condensate formation boosts PD-L1 transcription	Promotion	[108]
Neddylation	Acute promyelocytic leukemia (APL)	PML/RARa	Induce abberent LLPS and disrupt function of PML nuclear bodies to drive APL	Inhibition	[109]

## Deregulated phase separation in cancer

**Fig. 4:**
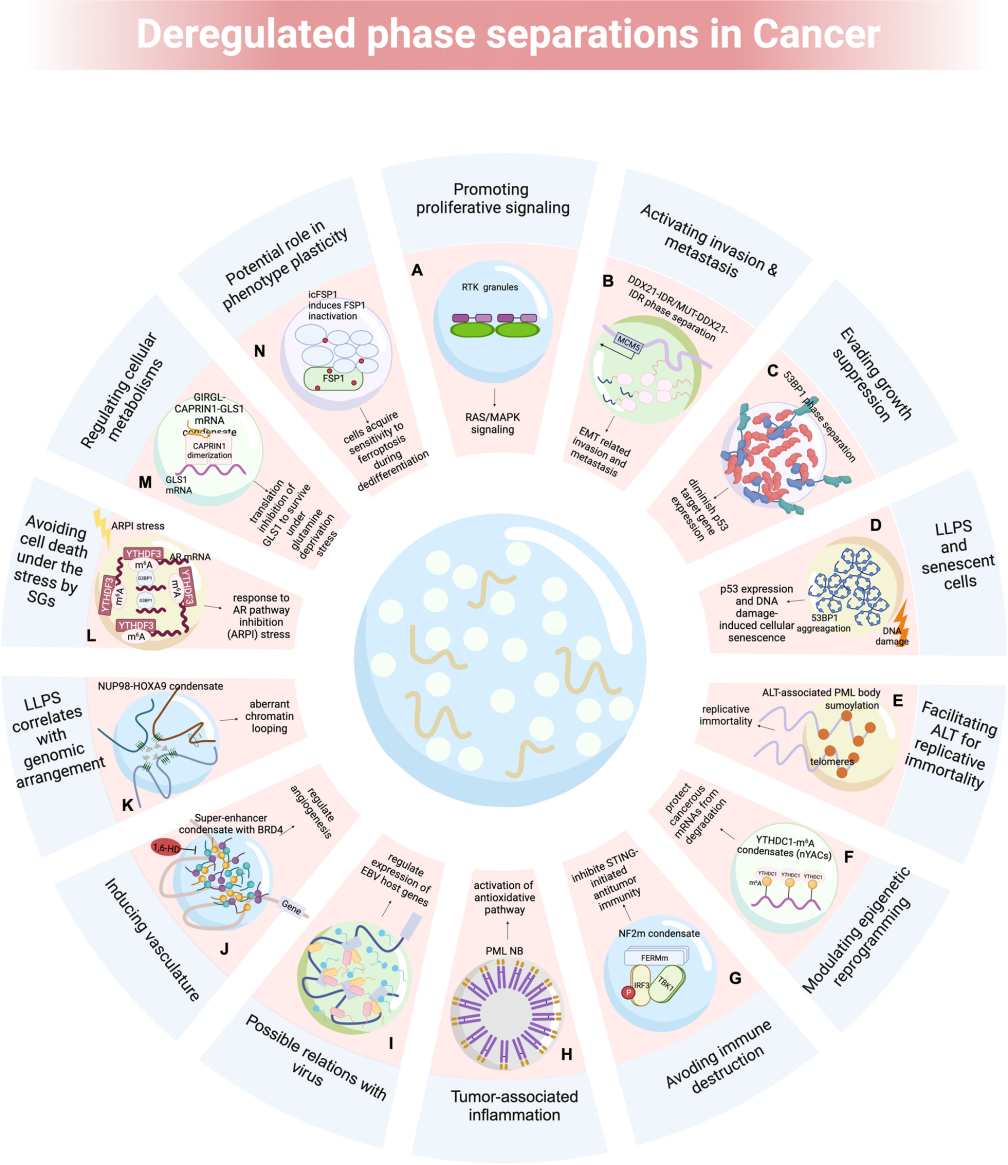
Summary of deregulated phase separations in cancer. **A** RTK granule formations activate RTK/MAPK signaling pathways to promote tumor proliferation. **B** DDX21phase separation activates MCM5, facilitating EMT signaling and modulating metastasis of colon cancer. **C** LLPS of 53BP1 diminish downstream targets of p53 to evade growth suppressions. **D** The accumulation of 53BP1 in the nuclear foci is enhanced after DNA damage, activating p53 and regulating cellular senescence. **E** SUMO ALT-associated PML bodies on the telomeres facilitate the replicative immortality of cancer cells. **F** Nuclear condensates (nYACs) generated through the LLPS of YTHDC1 (binding with m6A-mRNA) are significantly increased in AML cells. **G** Mutations in the FERM domain of NF2 (NF2m) robustly inhibited STING-initiated antitumor immunity by forming NF2m-IRF3 condensates. **H** PML nuclear bodies (NBs) serve as comprehensive ROS sensors associated with antioxidative pathways. **I** EBNA2 becomes part of BMCs and regulates EBV gene transcriptions. **J** BRD4 forms condensates with SEs to regulate angiogenesis. **K** NUP98-HOXA9 fusion proteins attenuate aberrant chromatin organizations. **L** m^6^A-modified androgen receptor (AR) mRNA phase separated with YTHDF3 responds to AR pathway inhibition (ARPI) stress in prostate cancers. **M** LLPS of GIRGL-CAPRIN1-GLS1 mRNA suppresses GLS1 translation and adapts to an adverse glutamine-deficient environment. **N** icFSP1 induces FSP1 condensates to trigger ferroptosis in the dedifferentiation of cancer cells

Emerging evidence has robustly revealed that aberrant BMCs are involved in various biochemical processes in human diseases and various oncogenic signaling pathways [19] (Table 3). Next, we review the role of LLPS in tumors based on several hallmarks (Fig. 4).

**Table 3:** Oncogenic signaling assosciated condensates that were involved in LLPS

Signaling Pathway	Cancer type	Biomolecule/ condensate	Biological role	Ref
EGFR/RAS signaling	Lung cancer	EGFR condensates	Regulating pro-tumor activation of Ras	[110, 111]
KRAS signaling	Lung cancer	EML4-ALK condensates	Modulating the KRAS signaling pathway, amplifying the oncogenic potential of this cascade, ultimately leading to dysregu- lated cellular proliferation and survival	[112, 113]
JAK-STAT3 signaling	Lung cancer	EZH2/STAT3	Myristoylation modification of EZH2 enables its phase separation, compartmentalize STAT3 within the condensates and leads to the sustained activation and enhanced transcriptional activity of STAT3	[113]
PI3K-AKT-mTOR signaling pathway	Lung cancer	stress granule	dynamically interacting with a key component of lung oncogenic pathway, mTOR and its regulators, influencing its localization, activity, and downstream signaling	[114]
Hippo signaling pathway	Pan-cancer	YAP, TAZ, TEAD	Undergoing LLPS, accumulating in the nucleus coregulator with increased activity in various cancers	[68, 115]
	Hepatocellular carcinoma	G6PC (glycogen compartments)	YAP signaling activation	[116]
	Hepatocellular carcinoma	YAP/TEAD transcriptional condensates	Acting as signaling hubs for the tumor microenvironment	[117]
	Hepatocellular carcinoma	Laforin-Mst1/2 condensates	Increasing hepatocarcinogenesis	[116]
p53 signaling	Pan-cancer	p53, 53BP1	53BP1 can form phase separation droplets, which enrich tumor suppressor protein p53. Cancer-associated mutation of p53 can accelerate the protein aggregation and amyloid formation by destroying the folding of p53 core domain	[118, 119]
Wnt/β-catenin signaling	Breast and prostate cancer	DACT1	WNT signaling inhibition	[120]
TGF-β signaling	Colorectal cancer	SMAD3	forming nuclear foci when the signaling pathway is activated	[121]
cAMP/PKA signaling	Atypical liver cancer fibrolamellar carcinoma	DnaJB1-PKAcat fusion	Tumorigenic cAMP signaling	[122]
	Hepatocellular carcinoma	RIα condensates	Promoting cell proliferation and transformation	[122]
RAS signaling	Pan-cancer	EML4-ALK fusion	RAS signaling overactivation	[123, 124]
	Pan-cancer	CCDC6-RET fusion	RAS signaling overactivation	[123, 124]
	Pan-cancer	LAT, GRB2, SOS	Activating Ras in tumour development	[125]
MAPK signaling	RTK-driven human cancer	SHP2	Stimulation of downstream MAPK signaling pathways and ERK1/2 activation	[126]
Wnt/β-Catenin signaling	Colorectal cancer	Destruction complex	Regulating development and stemness	[127]
NRF2/NF-κB signaling	Lung cancer	p62 bodies	Accelerating cancer development	[128]
NF-κB pathway signaling	Virus-associated cancer	p65/inclusion body	The trapped p65 (subunit of NF-κB) by phase separation of viral replication machinery cannot translocate into the nucleus to activate the downstream transcription of proinflammatory cytokine genes and other antiviral genes	[129]
cGAS-STING signaling	Pan-cancer	NF2m-IRF3 condensates	Regulating tumor immunity	[130, 131]
IL-6/STAT3 signaling	Hepatocellular carcinoma	Paraspeckles	IL-6/STAT3 signaling promotes paraspeckles formation, which favors overactivation of STAT3	[132]

### LLPS promotes the proliferation of cancer cells

Cancer cells can undergo unrestricted division [20, 133–136], which can occur through gene mutations that activate oncogenic receptor tyrosine kinases (RTKs) and the downstream MAPK signaling involving RAS proteins.

Adaptor proteins involved in RTK and RAS signaling, such as LAT, GRB2 and SOS, undergo phase separation during RTK activation [137]. This phenomenon increases the interaction time between SOS and RTK/RAS, providing a mechanism for kinetic proofreading during RTK activation [125, 138] and preventing the spontaneous membrane localization of SOS, and the downstream activation of RAS. Interestingly, carcinogenic RTK mutations resulting from chromosomal rearrangements cause the loss of membrane localization but not its ability to stimulate downstream pathways. Mechanically, these condensates can assemble the RAS-activating complex GRB2/SOS1, which activates the RAS-MAPK signaling in a membrane-independent manner [123]. Moreover, RTK fusion oncoprotein granules enable the activation of RTK signaling [123, 139]. The close binding to RTK oncoprotein condensates allows GRB2 to concentrate key downstream molecules, achieving the constitutive activation of RAS-MAPK signaling in cancer cells (Fig. 4A). Therefore, BMCs provide a new method for modulating cancer-promoting signaling in a spatially restricted manner.

### LLPS promotes the metastasis of tumors

The ability to invade and metastasize allows the tumors to develop distantly, and the epithelial–mesenchymal transition (EMT) programs are commonly involved [140]. Activated by EMT, the transcription coactivators YAP and TAZ facilitate metastasis [141, 142]. Hu et al. found that YAP fusion proteins undergo LLPS in the nucleus and that the IDR provided by the partner of YAP is required for assembly. This aggregation promotes the YAP/TAZ-specific transcriptions and attenuates metastasis [68]. Similarly, another study revealed that the phase separation of DDX21 activates MCM5, thus triggering EMT signaling and modulating the colon cancer metastasis (Fig. 4B)[143]. Besides, SGs are also involved in malignant invasion and metastasis. EMT markers Cadherin, Vimentin, Snail and Slug are suppressed under SG core component G3BP1 depletion, implying the role of G3BP1 in tumor metastasis [144]. Moreover, G3BP modulates mRNA stability under stress conditions and facilitates the invasion of cancer cells [145]. These carcinogenic mechanisms provide new explanations for tumor metastasis, as well as the inspiring ideas for models of cancer progression regulation by the BMCs.

### LLPS helps evade tumor growth suppression, regulate the aging process, and achieve replicative immortality of tumor cells

Cancer cells not only promote their growth but also modify tumor-suppression pathways [20]. By inhibiting tumor suppressors such as SPOP, p53, and RB1 [146–148], cancer cells escape intrinsic growth limitations. P53, one of the most well-known tumor suppressors, inhibits tumorigenesis via transcriptional activation, which leads to the disorders of apoptosis, cell cycle, and cell senescence. Tumor-associated stress significantly triggers p53 aggregation [149–154]. These findings demonstrate that the disruption of particular BMCs may cause cancer (Fig. 4C). Further studies are needed to validate this approach with other tumor suppressors and to test its potential applications.

Cellular senescence is considered an anticancer mechanism that maintain homeostasis and is associated with cell cycle arrest. The initiation and maintenance of cellular senescence rely on the frequent damage to the P53/Rb signaling pathway. Increased accumulation of 53BP1 in the nuclear foci after DNA damage can activate p53 and has recently been shown to regulate the cellular senescence via LLPS (Fig. 4D) [155].

Cancer cells can overcome the cell senescence and death via telomerase or alternative methods for lengthening telomeres (ALT) [156–158]. Multivalent interactions between SUMO and SUMO-interacting motifs were observed in the formation of ALT-associated PML bodies on telomeres in cancer stem cells (Fig. 4E) [159]. The fusion of PML bodies enables the clustering of telomere elements and the recruitment of DNA helicases, and other molecular machinery to extend the length of telomeres [160]. This finding suggests that cancer stem cells achieve replicative immortality through the unchecked cell division, and that this process is associated with LLPS.

### LLPS modulates epigenetic reprogramming of various BMCs

Common epigenetic modifications include histone modifications, DNA methylation, and RNA interference [161, 162]. Interactions between epigenetic modifications and their corresponding reader proteins also exhibit polyvalent interactions. M^6^A, known as the most common mRNA modification [163], alters the mRNA structure and interacts with multiple other mRNA modifications and proteins. This modification facilitates YTHDF protein phase separation, further contributing to the forming of various RNA–protein granules, including P bodies and SGs [74, 164]. In addition, YTHDC1 can undergo LLPS in the nucleus by interacting with m^6^A-modified mRNAs. This interaction results in the formation of nuclear YTHDC1-m^6^A condensates (nYACs), which are significantly enhanced in acute myeloid leukemia (AML) cells (Fig. 4F) [105].

### LLPS helps cancer cells escape immune destruction and participate in tumor-associated inflammation

The immune system employs the RLR-MAVS and cGAS-STING signaling pathways for protection against microbial invasion and support tumor immune surveillance [165–167]. However, tumors often escape immune clearance surveillance. Recent findings by Meng et al. revealed that neurofibromin 2 (NF2) facilitates innate immunity by eliminating tank-binding kinase 1 (TBK1) activation. It is the missense mutations in the FERM domain of NF2 (NF2m) that robustly inhibit the STING-initiated antitumor immunity via the NF2m-IRF3 condensates formations (Fig. 4G), suppressing the TBK1 activation [130]. This offers novel insights into NF2-related cancer treatments.

Notably, inflammation often plays a dual role in cancer. Overproduced in various inflammatory tissues, the reactive oxygen species (ROS) may accelerate the genetic mutations of cells, making them more aggressive and malignant [168]. However, recent research indicates that the PML NB may function as a sensor for ROS in two ways: protecting cancer cells from excessive ROS or promoting ROS-induced apoptosis (Fig. 4H). Given the lack of in-depth research in this field, further tumor microenvironment exploration is required to understand these processes fully.

Tumor-associated viruses, such as human papillomavirus, Kaposi sarcoma herpesvirus, and Epstein–Barr virus (EBV), influence tumor progression through LLPS [169–171]. In EBV proteins such as EBNA2 and EBNALP, LLPS regulates host gene expression, forming biomolecule condensates at Runx3 and MYC SE sites to regulate viral and cellular gene transcription (Fig. 4I). Further, the LLPS of EBNA2 can influence the alternative splicing of the pre-MPPE1 gene in cancer [170].

### LLPS induce vasculature of the tumors

Vascularization, also known as angiogenesis, is crucial for supplying tumors with nutrients and oxygen for growth. Vascular endothelial growth factor (VEGF) is the leading factor responsible for rapid nutrient supply. Mounting evidence has indicated a correlation between BMC formation and angiogenesis. For example, the constitutive expression of the transcription factor (TF) MYC in metastasizing cells can lead to VEGF transcription by potentially forming phase-separated transcription condensates, promoting promotes angiogenesis [172]. Similarly, the use of 1,6-hexanediol, an inhibitor of LLPS, has recently been shown to regulate angiogenesis by inhibiting cyclin A1-related endothelial functions as well as condensates with BRD4, indicating that targeting condensates can block critical reactions (Fig. 4J) [173, 174].

### Genomic arrangements initiate LLPS

Genomic instability contributes to tumor progression. Genomic translocations and rearrangements can lead to the fusion between the IDR of one protein and the DNA- or chromatin-binding domain of another [175]. This fusion acts as a TF, eliciting LLPS and attracting additional partners to initiate transcriptional programs that ultimately contribute to tumorigenesis. A typical example is the NUP98 fusion oncoprotein (FO), which occurs in 50% of patients with chemotherapy-resistant AML [176–179]. FOs demonstrate that malignancies establish cancerous TF condensates [83, 180, 181] and attenuate aberrant chromatin organization (Fig. 4K).

### LLPS of SGs assist in avoiding cell death of cancer cells under the stress

Cancer cells can escape apoptosis by forming SGs (a form of MLOs) when exposed to extreme conditions, such as high temperatures, toxins, mechanical damage, or other stresses. For example, the Y-box binding protein 1 (YB-1) interacts with the 5'-untranslated region (UTR) of G3BP1[182], leading to the increased expression of G3BP1 and SGs, which is elevated in human sarcomas [183–185]. Consequently, these cancer cells survive hyperproliferation, chemotherapy and other various stressful conditions. Additional studies on prostate cancer have demonstrated that the m^6^A-modified androgen receptor (AR) mRNA phase separated with YTHDF3, while the unmodified AR mRNA phase separated with G3BP1 to survive AR pathway inhibition stress (Fig. 4L)[186]. Collectively, SGs may serve as novel targets for cancer biology investigations.

### LLPS regulates cellular metabolisms of cancer cells

Malignant cells undergo metabolic reprogramming [187], thereby attracting considerable interest in tumor-related research in the past decades [188]. For example, the reduction of glutaminase-1 (GLS1) enables cancer cells to survive under prolonged glutamine deprivation stress [189, 190]. Wang et al. reported that the lncRNA GIRGL promotes the LLPS of GIRGL-CAPRIN1-GLS1 mRNA to suppress GLS1 translation, thus adapting to an adverse glutamine-deficient environment (Fig. 4M)[191]. CAPRIN1, an RNA-binding protein involved in the SG formation via LLPS, plays a role in this metabolic adaptation. Therefore, alteration of cell adaptation to an adverse metabolic environment is possible by targeting condensates.

#### Potential role of LLPS in the phenotypic plasticity of tumorigenesis

Tumor cells often exhibit phenotypic plasticity to evade terminal differentiation. This plasticity includes the dedifferentiation, the differentiation inhibition, and the transdifferentiation [20]. During dedifferentiation, specific malignant cells become sensitized to ferroptosis [192–194], a form of cell death. Nakamura et al. [195] first demonstrated that the novel FSP1 inhibitor, icFSP1 impairs cell proliferation and induces FSP1 condensation to trigger ferroptosis in cancer cells (Fig. 4N). This highlights the role of iron in tumor progression and the dependence of cancer cells on iron in drug-resistant states.

## Clinical applications of LLPS in oncologic fields

### Potential of LLPS in cancer treatments

Considering that various regulatory mechanisms of LLPS are closely associated with tumorigenesis, it is imperative to explore therapeutic approaches against abnormal LLPS. These strategies can be categorized into three main approaches (Table 4).

**Table 4:** Summary of strategies and drugs that use phase separation to intervene in tumorigenesis and progression

Targeting strategy	Drug/molecules	Tumor types	Associated protein/condensate	Mechanism of action	References
Disruptions of the BMCs’ formations	IIA4B20, IIA6B17, mycmycin-1/2	Pan-cancer	Myc	Preventing the Myc/Max dimerization inhibit Myc-induced malignant transformation	[196]
	YK-4–279	EWS	EWS-FLI1 fusion	Binding to the IDR of the oncogenic transcription factor EWS-FLI1 and prevents the interaction between EWS-FLI1 and RNA helicase A, thereby slowing down EWS cell growth	[197]
	elvitegravir	Lung cancer	SRC1	Directly binding to the highly disordered SRC1 and effectively inhibit YAP oncogene transfer by disrupting liquid–liquid separation in SRC1/YAP/TEAD condensates	[117]
	C108	Breast cancer	G3BP2 (SG core component)	Diminishing the role of SG core component G3BP2 in breast cancer initiation and improve the efficacy of chemotherapy drugs	[198]
	2142–R8 peptide	Pan-cancer	KAT8–IRF1 condensates	disrupt the formation of KAT8–IRF1 condensates, subsequently suppressing PD-L1 expression and enhancing antitumor immunity in vitro and in vivo	[198]
	BAY 249716	Pan-cancer	p53	Inducing condensate formation of DNA-binding defective mutants; dissolve nuclear condensates of structural mutants; covalent binders	[199]
	BAY 1892005				
	Avrainvillamide	Aml	NPM1	Restoring nucleolar localization of cytoplasmic NPM1 mutant; covalent binder	[200]
	SHP099	RTK-driven human cancer	SHP2	Stabilizing SHP2 in an auto-inhibited conformation and suppressing RAS–ERK signalling to inhibit the proliferation of receptor-tyrosine-kinase-driven human cancer cells	[201]
	ET070	RTK-driven human cancer	SHP2	Inhibiting the phase separation ability of SHP2 mutants by locking SHP2 in the “off” conformation	[126]
	JQ1	Breast cancer and colon cancer	BET family of bromodomain proteins	Partitions into transcriptional condensates; dissolving MED1 nuclear condensates	[202]
	EPI-001	Prostate cancer	Androgen receptor	Dissolving androgen receptor-rich transcriptional condensates	[203]
	Leptomycin B	Leukemia	CRM1	Inhibiting formation of aberrant NUP98–HOXA9 transcriptional condensates	[204]
	Ribavirin	Prostate cancer	OCT4/AR/FOXA1, OCT4/NRF1	Inhibiting the formation of OCT4-AR axis by modulating OCT4 condensates in the nucleus	[205]
	Tin (IV) oxochloride-derived cluster	Pan-cancer	IDR of TAF2 in TFIID	Disrupting transcription initiation by selectively impairing the function of TFIID	[206]
	PRIMA-1; ReACp53	Ovarian carcinoma	p53 mutants	Induction of cell cycle arrest in cancer cells with mutant p53 by restoring the native conformation of aggregated mutant p53 proteins	[207]
	PCG	Breast cancer	IDR of BRD4	Suppression of BRD4-dependent gene transcription	[208]
	bis-ANS	Colon cancer	LCD of TDP-43	high concentrations of bis-ANS inhibit TDP-43 condensate assembly, whereas low concentrations facilitate the formation of liquid droplets	[209]
Modifications of PTMs and physicochemical conditions	SI-2	Multiple myeloma	SRC3/NSD2 condensate	Phase separation of SRC3 mediated by histone methyltransferase NSD2 leads to resistance to bleitinib in multiple myeloma, whereas the inhibitor SI-2 Inhibits formation of drug-resistant SRC3/NSD2 condensates and improves the therapeutic efficacy of bleitinib	[210]
	Olaparib	Pan-cancer	PARP1/2 DNA repair condensate	Inhibiting PARP1/2 and thus interferes with the formation of PARylation related DNA repair condensates	[211]
	GSK-J4	Osteosarcoma	HOXB8/FOSL1 CRC	The H3K27 demethylase inhibitor GSK-J4, inhibits the CRC phase separation and results in metastasis suppression and re-sensitivity to chemotherapy drugs	[212]
	icFSP1	Melanoma and lung cancer	hFSP1	Inducing phase separation of myristoylated hFSP1, thus promoting ferroptosis and inhibit tumor proliferations	[195]
	GSK-626616	Pan-cancer	DYRK3	Inhibit PRAS40 phosphorylation and restrain mTORC1 signaling in SGs	[213]
	JQ1	AML	BRD4	Release the Mediator complex from SEs	[214]
	SGC0946	MLL leukemia	DOT1L	Inhibit histone H3K79 methylation and histone H4 acetylation	[215]
	THZ1	Pan-cancer	CDK7	Inhibit RNAPII phosphorylation	[216]
	THZ531	Pan-cancer	CDK12 and CDK13	Inhibit RNAPII phosphorylation	[217]
Drug interventions and distributions of the dynamics of condensates	Cisplatin	Breast cancer and colon cancer	MED1 transcriptional condensates	Partitions into transcriptional condensates; dissolves MED1 and BRD4 nuclear condensates	[202]
	Tamoxifen	breast cancer and colon cancer	Estrogen receptor	Seletively partitions into transcriptional condensates	[202]
	mitoxantrone	breast cancer and colon cancer	Estrogen receptor	Seletively partitions into transcriptional condensates	[202]
	PML-retinoic acid receptor α	APL	PML bodies	Hindering the assembly of PML bodies and result in the suppression of differentiation genes. Successful APL treatment involves the restoration of PML nuclear bodies using empirically discovered drugs	[109]

BMC, biomolecular condensate; EWS, Ewing sarcoma; IDR, intrinsically disordered region; SG, stress granule, RTK, receptor-tyrosine-kinase; CRC, core regulatory circuitry; MLL, mixed lineage leukemia; PML, Promyelocytic leukemia protein; APL, acute promyelocytic leukemia

#### Disruptions of the formation of BMCs

**Fig. 5:**
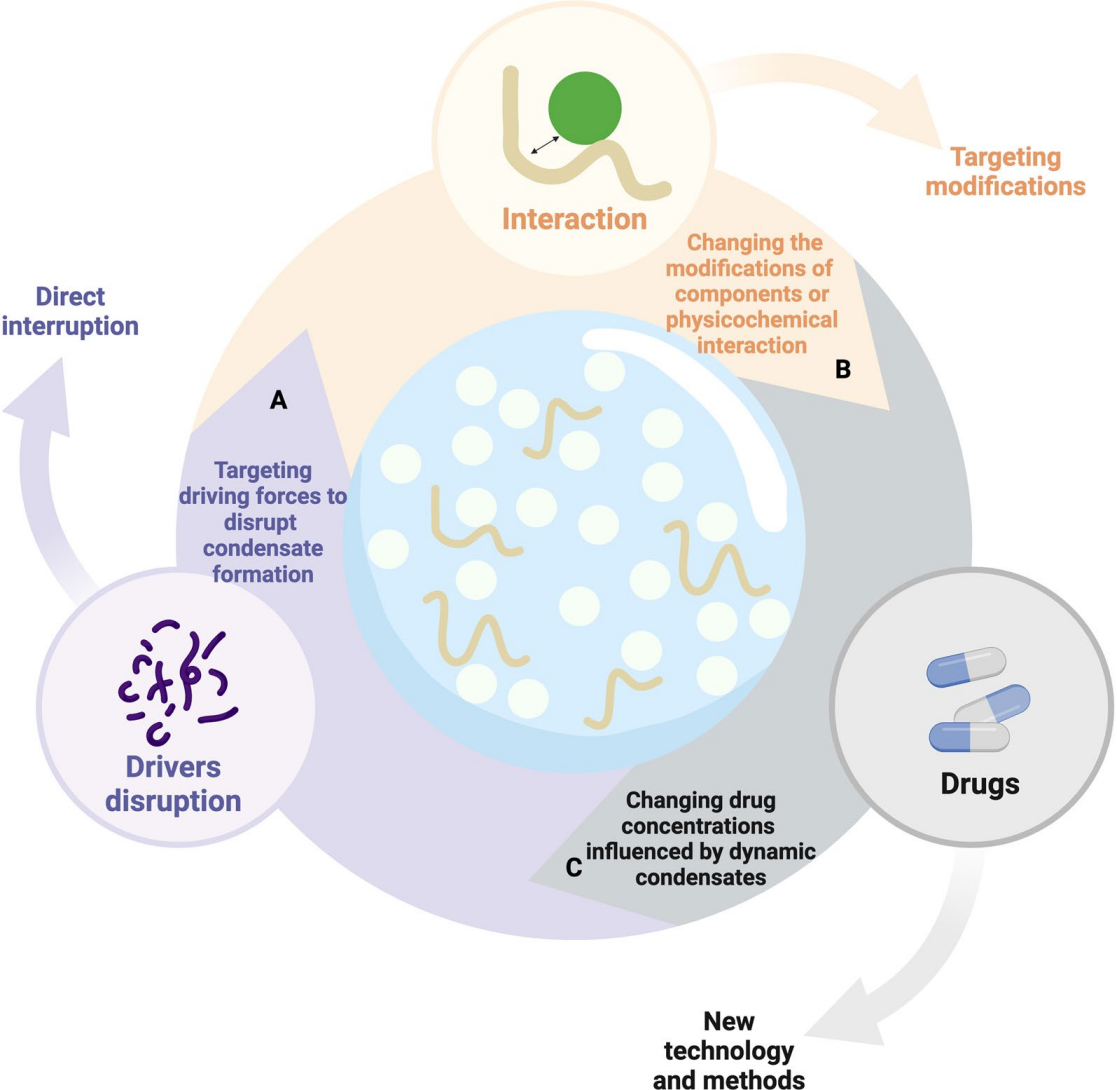
Potential approaches to developing new cancer treatments by regulating BMCs. **A** Targeting driving forces to disrupt condensate formation. **B** Changing the modifications of components or physicochemical interaction. **C** Drug concentrations influenced by dynamic condensates

The direct disruption of the driving force behind LLPS offers a straightforward approach (Fig. 5A). For example, certain drugs can intervene in the LLPS process by targeting IDRs of proteins. Notably, the anti-HIV drug elvitegravir directly binds to the highly disordered steroid receptor coactivator 1, effectively inhibiting oncogene YAP transcription by disrupting SRC1/YAP/TEAD condensates (Table 4) [117]. Similarly, Yu et al. reported that the nuclear translocation of YAP and LLPS are affected by IFN treatment in cancer cells. Therefore, interrupting the LLPS of YAP can inhibit cancer cell proliferation and enhance the immune response, indicating its potential as a predictive biomarker in immune checkpoint blockade [67]. Further, altering interactions between LCDs indirectly modulates the transcriptional subunits, thus offering a promising approach for targeting disease-causing TFs.

#### Modifications of PTMs and physicochemical conditions

As previously mentioned, certain post-transitional modifications and physiochemical conditions contribute to LLP dynamics (Fig. 5B). For example, nYACs protect mRNAs from degradation and strengthen the role of YTHDC1 in leukemogenesis, which inspires us to disrupt m^6^A to violate deleterious condensates[105]. Further, studies have reported that modulating PTMs in LLPS proteins is also significant [25, 96, 102, 218–221]. In the case of colon cancer, SENP1 has been reported to decrease RNF168 SUMOylation, inhibit nuclear condensate formation, and promote DNA damage repair (DDR) and drug resistance. Given these observations, strategies to curb the harmful effects of protein aggregation by influencing protein modifications warrant further investigation.

#### Drug interventions of the dynamics of condensates

Drugs can significantly influence the dynamics of the condensates, affecting their anticancer effects and potentially leading to drug resistance (Fig. 5C). For example, in luminal breast cancer, tamoxifen accumulates in MED1 condensates, preventing the incorporation of ERα into these condensates, partially inhibiting cancer progression. However, when MED1 is overexpressed, larger condensates dilute the drug concentration, ultimately leading to the development of resistance [202]. Several drugs, such as cisplatin, mitoxantrone, and THZ1, selectively partition into BMCs formed by MED1 (Table 4). Drug resistance can occur via selective partitioning into BMCs or changes in properties. Notably, cisplatin exerts its anticancer activity by dissolving SEs, indicating that changes in the condensate properties may improve therapeutic outcomes[202]. This finding highlights the potential of altering the properties of condensates to improve therapeutic outcomes. In some cases, promoting the formation of BMCs may have therapeutic effects. For example, in APL, fusion proteins of PML-retinoic acid receptor α (RARA) hinder the assembly of PML bodies and result in the suppression of differentiation genes. Successful APL treatment involves the restoration of PML nuclear bodies using empirically discovered drugs (Table 4) [222].

### Roles of LLPS in vesicular trafficking and drugs’ delivery

Although LLPS and traditional vesicles are two different concepts with distinctive definitions, the vesicular trafficking role of LLPS is still rarely described and attractive. Conventional approaches typically utilize nanoscale carriers that are confined within the compartments of the intranuclear body. Nevertheless, recent findings have demonstrated that micron-scale polypeptide clusters, formed through phase separation, possess the ability to traverse the cell membrane via a non-canonical endocytic pathway. These clusters undergo glutathione-induced release of their cargo and exhibit the capacity to rapidly incorporate various macromolecules into microdroplets, such as RNA, small peptides and enzymes [223]. Loaded with polysomes, they can provide new approaches for vaccine carriers based on mRNAs and intracellular transportations for cancer treatments.

Likewise, as previously mentioned, droplets of drugs formed by LLPS can unexpectedly raise the inner drug concentration up to 600 times higher than that outside the condensate [202]. Furthermore, MED1 predominantly acts on oncogene promoters, thereby enabling cisplatin to ultimately target the corresponding DNA through its toxic platinum atoms, effectively assaulting the vital components of the cancer cells. Besides, the phosphopeptide KYp has been observed to induce LLPS level at the cell membrane, thus enhancing the permeation and internalization of the peptide drug [224]. KYp has the ability to interact with alkaline phosphatase, resulting in the dephosphorylation and in situ self-assembly at the cell membrane [224]. The process induces the aggregation of alkaline phosphatase and the separation of proteolipid phases at the membrane, ultimately enhancing membrane leakage and facilitating the entry of the peptide drug. These great discoveries provide inspirations for designing drug delivery systems and more similar ideas are worth exploring.

## Conclusions and future perspectives

In the past decades, crucial advances have been made in figuring out the role of LLPS in a variety of cellular processes and biological functions. Since the update of the new version of “Hallmarks of cancer 2022”, cancer hallmarks and their enabling characteristics help distill the oncogenic complexity into an evidently logical science, which have been gradually proven to be closely associated with LLPS. In this review, we summarize the mechanism of LLPS formations, recent discoveries and the individual role of LLPS in oncology. These findings collectively reveal its vital role in solving undruggable targets and multiple clinical problems.

The role of specific proteins and post-translational mechanisms in the formation and regulation of LLPS are being investigated. These efforts aim to identify abnormal conditions and gain insights into the mechanisms regulating the formation of the condensates. These studies have already begun to help find new strategies for targeting disease-related condensates. Notably, while previous drugs were designed to inhibit each protein directly, LLPS offers a novel and unexpected possibility of interfering with the pathological process and does not necessitate targeting each protein individually. This approach achieves disruption of the condensates formed by IDRs of TFs.

Despite the steady progress in targeting BMCs using LLPS, several fundamental questions need to be answered. For example, what are the functional differences between LLPS-formed assemblies and typical protein complexes? What factors contribute to dynamic condensation and decondensation, and how do different BMCs communicate in vitro and in vivo? Moreover, the role of PTMs in tumorigenesis requires further exploration (Table 5). Clarifying these aspects will improve our understanding of the conversion of physiological into pathological condensates in cancer. Future research will require collaborative efforts, innovative approaches, and a holistic approach to studying cancer-associated LLPS, which may lead to novel anti-tumor therapies directly targeting BMCs.

**Table 5:** Outlook and reflection on the future of the field

Critical issues in the current development of oncogenic LLPS	Outlook and reflection on the future/ possible solutions to the questions
What are the functional differences between LLPS-formed assemblies and typical protein complexes? What factors contribute to dynamic condensation and decondensation, and how do different BMCs communicate in vitro and in vivo?	The target protein molecules and signaling pathways discovered through LLPS method are a class of molecules that can form condensates spontaneously due to their own unique properties or under different environmental conditions. LLPS is essentially an energy saving process in the organisms. Further functional differences between LLPS‐formed assemblies and canonical protein complexes deserve investigations
Is there other function of PTMs in tumorigenesis and tumor progressions?	Further studies on phase separation on the basis of proteomics and PTMs are needed
Detections of BMCs/ MLOs in tumor samples and clinicopathologic associations with cancer patients are deficient	Clinicopathologic tests should be involved in further studies
How do environment conditions inducing condensate assemblies being applied to clinical practice?	Perhaps changing the environment conditions can dynamically alter the condensation and decondensation of the BMC, which will make sense in drug deliveries. A greater understanding of the opportunities for targeting LLPS condensates in the pharmaceutical intervention should be obtained
Is there any new convenient method to probe and control (induce, dissolve, or tune) the endogenous condensates?	The partitioning of anticancer drugs in subcellular condensates is also dominant for drug efficacy. According to this characteristic, we can detect the distribution of drugs in cells or by linking drugs to molecules that can specifically aggregate in liquid droplets
How to make use of LLPS to enhance the efficiency of drugs in clinical practice?	

## Data Availability

Not applicable.

## Abbreviations

ALT: Alternative lengthening of telomeres
ALK: Anaplastic lymphoma kinase
AML: Acute myeloid leukemia
AR: Androgen receptor
AS: Alternatively spliced
APL: Acute promyelocytic leukemia
BMCs: Biomolecular condensates
DDR: DNA damage repair
EBV: Epstein–Barr virus
EMT: Epithelial–mesenchymal transition
FO: Fusion oncoproteins
GLS1: Glutaminase-1
IDR: Intrinsically disordered regions
LCDs: Low-complexity domains
LLPS: Liquid-liquid phase separation
MLOs: Membrane-less organelles
NB: Nuclear body
NF2: Neurofibromin 2
N-WASP: Neural Wiskott—Aldrich Syndrome
PRM: Proline-rich motif
PTM: Post-translational modification
RARA: Retinoic acid receptor α
RBPs: RNA-binding proteins
ROS: Reactive oxygen species
RTKs: Receptor tyrosine kinases
SEs: Super-enhancers
SERBP1: SERPINE1 mRNA-binding protein 1
SG: Stress granule
SPOP: Speckled POZ protein
TF: Transcription factor
UTR: Untranslated region
VEGF: Vascular endothelial growth factor
